# Warfarin-induced skin necrosis: a rare condition

**DOI:** 10.4314/gmj.v54i4.10

**Published:** 2020-12

**Authors:** Josephine Nsaful, Yaw Ofori Adjei, Florence Dedey, Nelson Agboadoh, Edem Anyigba, Warigbani Pieterson

**Affiliations:** 1 Department of Surgery, University of Ghana Medical School, Accra, Ghana; 2 Department of Medicine, Korle Bu Teaching Hospital, Accra, Ghana; 3 Department of Surgery, Korle Bu Teaching Hospital, Accra, Ghana; 4 National Reconstructive Plastic Surgery and Burns Centre, Korle Bu Teaching Hospital, Accra, Ghana

**Keywords:** warfarin, skin necrosis, anticoagulation, Ghana

## Abstract

Warfarin induced skin necrosis is a rare debilitating and, in some cases, life-threatening complication. A 47-year-old male on life-long anticoagulation omits his medication and develops extensive skin necrosis of the left leg complicated by acute renal failure three days after restarting warfarin. Investigations reveal possible Protein S deficiency which is known to be a predisposing condition. Various mechanisms have been proposed as the underlying cause. He was managed on heparin, wound debridement and skin grafting. Warfarin was restarted concurrently with heparin. Knowledge of this complication will enable timely diagnosis and treatment.

**Funding:**

None declared

## Introduction

Warfarin is a widely used oral anticoagulant for a variety of conditions including deep vein thrombosis, pulmonary embolism and in patients who have prosthetic heart valves. Side effects of warfarin use include common ones such as bleeding and rare ones such as skin necrosis. Warfarin induced skin necrosis (WISN) was first recognised in 1952 in the Netherlands^1^ but was previously described in 1943 without linking it to anti-coagulant therapy.^2^ It is a rare debilitating and in some cases life threatening complication of anticoagulant therapy. Knowledge of this paradoxical phenomenon is important as it could be mistaken for acute arterial thrombosis, necrotising fasciitis, cellulitis, phlegmasia cerulea dolens, disseminated intravascular coagulopathy with purpura fulminans or cutaneous manifestation of an autoimmune disease.^3^, ^4^, ^5^ It should be recognised and treated promptly. We present one such case who presented in a critically ill state.

## Case Report

A 47-year-old male was referred to the Emergency Room of Korle Bu Teaching Hospital (Accra, Ghana) from another facility where he had been admitted for over 48 hours. He was extremely dyspnoeic with orthopnoea, painful swelling and blisters of the left lower limb which started 3 days prior to presentation at the referring hospital. Patient gave a history of having had a thoracotomy for pulmonary embolism 11 years ago with insertion of an inferior vena cava filter.

He had been on warfarin 5mg daily since the surgery but did not go for reviews. He had missed 3 days of warfarin and as such restarted himself at a higher dose of 7.5mg daily 3 days prior to the onset of the above symptoms. A diagnosis of warfarin toxicity was made at the referral centre after an episode of epistaxis. Warfarin was stopped, IV vitamin K 10mg daily initiated and fresh frozen plasma given.

**Figure 1:**
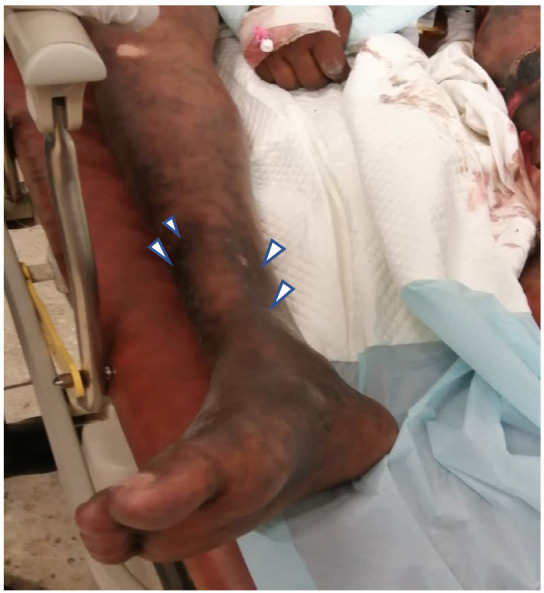
Right leg with ecchymotic patches (arrow heads)

**Figure 2:**
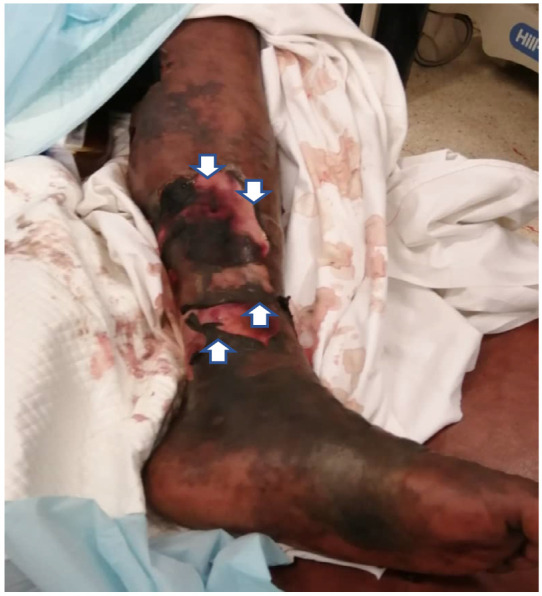
Ecchymotic patches on left leg with ruptured bullae (arrows)

At presentation, he was acutely ill and severely dyspnoeic with oxygen saturation of 92% on room air, blood pressure was 115/60mg and temperature 38.9°C. He had Kussmaul breathing, bronchial breath sounds and bilateral crepitations which were coarse over the right lung base. Both lower limbs had extensive ecchymotic patches with multiple large haemorrhagic bullae on the left (Figures 1 and 2) and one large bulla on the posterior aspect of the right knee. The left leg was cold relative to the right leg but regained its warmth the following day. There were numerous petechiae on the lower trunk and distal upper limbs which resolved over the next few days.

Significant findings in the initial laboratory investigations were urea 34mmol/L (2.1–7.1), creatinine 805 umol/L (62 – 106), and an estimated GFR (CKD-EPI) of 6ml/min, deranged liver enzymes, serum albumin 29g/L (39.7–49.5), INR 2.0 (1.0–1.25) and WBC 25.52 × 10^9^ (4.0–12.0) creatinine kinase 557IU/L (<190), serum calcium 1.91mmol/L (2.15–2.50).

Chest x-ray revealed consolidation of the right lower lobe and cardiomegaly. Accompanying echocardiogram revealed severe pulmonary hypertension, right ventricular and atrial enlargement with right ventricular failure, normal left ventricular contractility and left ventricular ejection fraction of 60%, there were no intracardiac thrombi. Hepatitis and HIV screens were negative. Samples were also taken for lactate dehydrogenase and thrombophilia screen. Initial diagnoses made were acute limb ischaemia from thromboembolic phenomenon, right heart failure from recurrent pulmonary embolism, acute kidney injury, sepsis, right lobar pneumonia and warfarin toxicity.

An urgent doppler ultrasound of the of the lower limbs revealed good arterial blood flow and there was no deep vein thrombosis. A final diagnosis of warfarin induced skin necrosis was made complicated by right heart failure and acute kidney injury.

The patient was started on subcutaneous Enoxaparin 40mg daily, IV Meropenem 500mg daily and oral Sildenafil 12.5mg daily. Other treatment was supportive. His renal function and general wellbeing improved after three sessions of dialysis done during the first week of admission. After which full treatment dose of subcutaneous Enoxaparin 100mg daily and IV Meropenem 500mg 8 hourly was continued. Blood cultures done were negative. Repeat echocardiogram at this time revealed evidence of previous pulmonary endarterectomy and tricuspid valve replacement, mild dilation of right chambers with mild right ventricular hypertrophy and normal left ventricular systolic function.

Protein C and Protein S taken initially were low 41% (70–140) and 49% (60–123) respectively. At this time this could have been due to the inhibitory effect of the warfarin on these naturally occurring anticoagulants. Repeat Protein C taken when patient had been off warfarin for two weeks had normalised 104% (70–140). However, Protein S was still depressed. This could be due to a deficiency, predisposing him to thrombotic diseases and to WISN. Anticardiolipin antibodies, Factor V Leiden and Antinuclear factor were negative.

**Figure 3:**
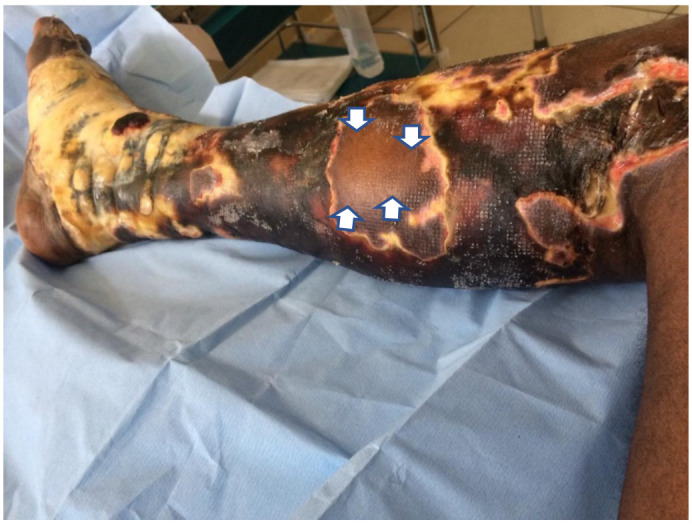
Full thickness skin necrosis with sparing of skin islands (arrows) on left leg

Skin necrosis on the left lower limb involved the entire leg up to the knee and foot sparing only part of the sole (Figure 3). The 4^th^ toe eventually became gangrenous and was amputated. There was a large necrotic patch with eschar on the posterior aspect of the right lower limb.

Alternate day dressings with normal saline were done allowing for demarcation of necrotic tissues. Pain was managed with opioids. Physiotherapy was initiated as soon as he was stable.

The patient first had debridement under spinal anaesthesia 3 weeks after admission. Wound swabs taken intraoperatively cultured *Pseudomonas aeruginosa.*

**Figure 4:**
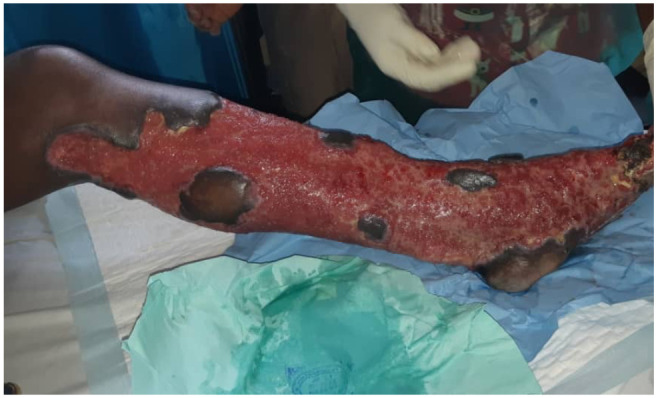
Fully granulated wound after dressing with nano crystalline silver

**Figure 5:**
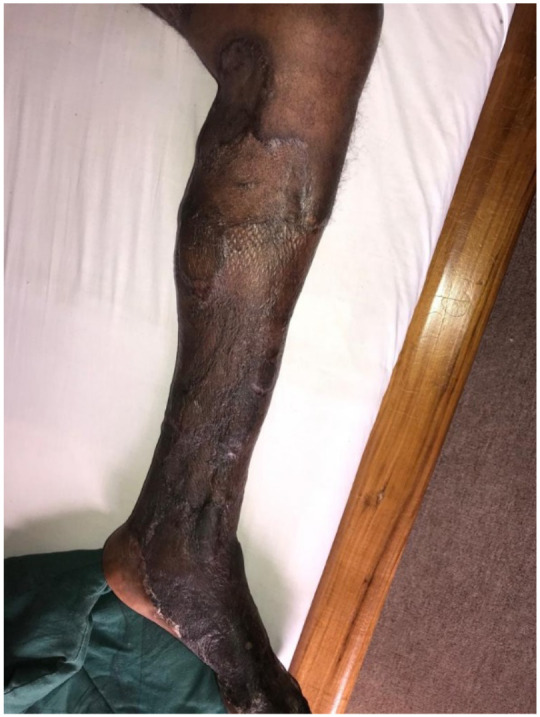
Good skin graft take after serial grafting

This was followed by a second debridement a week later and nano crystalline silver dressings started (Figure 4). Two sessions of split skin grafts were done to cover the left leg three weeks apart. He received a total of four pints of blood as haemoglobin had dropped to 8.7g/dl. He was readmitted for a third skin graft to cover the initial donor site harvested three months prior which had not healed. Six months after the initial injury patient's wounds were all healed (Figure 5) and he was mobilising without aid. He was restarted on warfarin by the cardiologist whilst on enoxaparin at a dose of 5mg daily and has a stable INR of 2.2 to 3.1 on the same dose.

## Discussion

WISN is a very rare side effect of warfarin use and estimated at 0.01% to 0.1% of patients on warfarin with about 300 cases reported worldwide.^3^,^6^,^7^ It is more common in females than men (4:1), usually affecting perimenopausal obese women but the reason is not apparent.^3^,^7^

The exact cause of WISN has not been properly established. It is believed that microvascular thrombosis is mainly responsible for the skin necrosis seen.^6^ The pathology may be multifactorial as others have proposed not only thrombosis but hypersensitivity, capillary haemorrhage and the direct toxicity from warfarin.^3^,^7^,^8^ There is very little proof to support these popular theories. Indeed, there are reported cases of WISN developing upon recommencement of warfarin in non-warfarin naïve patients.^9^,^10^ Certain conditions such as Protein C deficiency are believed to play a contributory role.

The anticoagulant effect of warfarin is due to its inhibition of vitamin K-dependent clotting factors II, VII, IX and X. However, it is also known to deplete the anticoagulants Protein C and S, which are also vitamin-K dependent. There is therefore an imbalance between anticoagulation and thrombosis with a tendency towards thrombosis as Protein C is inhibited rapidly together with factor VII then factor IX, X, protein S and II respectively deplete slowly.^1^,^11^,^12^ This can predispose the patient to thrombosis and is worse in patients who already have protein C deficiency. Other less common predisposing factors include Protein S deficiency, Factor V Leiden mutation, Antithrombin III deficiency, Antiphospholipid antibody syndrome (Anticardiolipin antibody) and Lupus anticoagulant.^3^,^13^ Several reported cases have however occurred in patients without any of these deficiencies.^14^,^15^

When biopsies are taken early histopathology shows deposition of fibrin in postcapillary venules and small veins.^16^,^17^

Typically, there are multiple microthrombi in the capillaries and venules of the dermis and subcutaneous tissue which results in ischaemic necrosis of the skin.^18^ There are subepidermal haemorrhages which clinically present as petechiae and result from rupture of the capillaries. These soon coalesce to form ecchymotic patches.^19^,^20^

Like most other case reports we came across, symptoms started as early as three days after warfarin was initiated.^3^,^6^,^17^ Though most cases present early in 1–10 days, delayed onset of symptoms have been reported as late as 3 years after onset of treatment.^14^ Preceding symptoms include paraesthesia, sensation of pressure and an erythematous flush.^3^ Petechiae develop and progress to large ecchymotic patches which blister as underlying skin necrosis develops. The site of necrosis varies but is common in fatty areas such as the thighs, buttocks, abdomen and commonly breasts in women^3^,^7^,^14^,^15^,^17^ Necrosis in the lower limbs as in this case is also common.^6^,^7^,^20^,^21^

The general principle of management of any adverse drug event is the withdrawal of the offending drug. However, in the instance of WISN, there is very little evidence that the withdrawal of warfarin affects the course of the ailment.^6^,^19^ Indeed the discontinuing, continuing and restarting therapy after an initial interruption does little to affect the established skin lesions or the emergence of new ones. Warfarin is stopped and therapeutic dose of heparin initiated, in this case enoxaparin was given. Vitamin K and fresh frozen plasma is recommended to reverse the protein C and S depletion.^3^,^6^,^15^ Protein C concentrates may be given in cases of deficiency.^6^

In this case wet saline wound dressings were done. Silver sulfadiazine, zinc creams and aluminium hydroxide ointments have also been used successfully till wound heals.^21^ Small lesions may respond to autolytic debridement with hydrogel and hydrocolloid dressings and heal by secondary intention.^15^ Unless skin necrosis is not extensive surgical debridement is usually done, twice in this patient followed by skin grafting. About 50% of cases need surgery and infrequently amputation of the extremity.^3^,^13^

Currently, there are no clear guidelines for the reintroduction of warfarin for patients who have suffered WISN and require long-term anticoagulation. It has been suggested that to prevent the development of WISN; large doses of warfarin should be avoided at the start of treatment to reduce the rapid decline of protein C. Rather gradual increments is recommended with heparin anticoagulation simultaneously to counter the transient procoagulant effect of warfarin until the desired INR is achieved.^13^

The caution here is skin necrosis from heparin induced thrombocytopaenia has also been documented and is grossly and histologically similar to WISN.^3^,^21^ For patients with WISN who require long term anticoagulation it may be advisable to switch from Warfarin to a Non-Vitamin K Antagonist such as dabigatran and rivaroxaban where available.^22^

## Conclusion

Knowledge of WISN is important for early diagnosis and timely treatment. To prevent this, patients should be started on low dose warfarin simultaneously with heparinisation and dose of warfarin gradually increased as required. Re-introduction of warfarin after WISN was safely done in this case.
